# Investigation of the effectiveness of Gross model-based cognitive emotion regulation training in the improvement of Love Trauma Syndrome, Hoping and Positive Affect Negative Affect among female students with love trauma

**DOI:** 10.5249/jivr.v15i2.1824

**Published:** 2023-07

**Authors:** Sayed Ali Sharifi Fard, Akbar Ata-Dokht, Sajjad Bashar Poor, Golnaz Ali Babaei

**Affiliations:** ^*a*^ Faculty of Educational Sciences and Psychology, University of Mohaghegh Ardabili, Ardabil, Iran.; ^*b*^ Department of Psychology, University of Mohaghegh Ardabili, Ardabil, Iran.

**Keywords:** Love trauma, Hoping, Positive affect, Negative affect, Emotion cognitive regulation, Gross model

## Abstract

**Background:**

Emotional sadness caused by the experience of the loss of a romantic relationship can lead to love trauma syndrome, which includes a set of psychopathology symptoms. The present study was also conducted to investigate the effectiveness of cognitive emotion regulation training based on the Gross model in improving the love trauma syndrome, hoping and positive affect negative affect among female students with love trauma.

**Methods:**

The research method was experimental (pre-test and post-test with the control group). The statistical population was all the female students with love trauma at the University of Mohaghegh Ardabili and 34 participants (17 participants in each group) were selected by purposive sampling. Also, in order to determine the target sample from the Love Trauma Syndromes Inventory (LTSI-10), to implement the intervention of the cognitive emotion regulation program based on the Gross model, and to measure dependent variables in addition to the LTSI, Positive Affect Negative Affect Scale (PANAS-20) and Miller Hope Scale (MHS-48) were used. Multivariate analysis of covariance (MANCOVA) was also used for data analysis.

**Results:**

The results showed that the assumptions (homogeneity of covariances and variances) are maintained. Also, the effect of group membership shows the significant impact of the intervention on the love trauma syndrome (p less than 0.01), negative affect (p less than 0.01), hope (p less than 0.01), and no effect on positive affect (p greater than 0.05).

**Conclusions:**

Therefore, after identifying individuals with love trauma, the intervention of cognitive emotion regulation can be done on them in a group to reduce the symptoms of psychological harms in them and also to cognitive strategies, and to equip them for problem solving or compatibility.

## Introduction

Rempel and Burris^1^ define love as a relationship that is neither a form of behavior nor an emotion, but they define love as a "motivational state whose purpose is to preserve and promote the well-being of the valuable object that is the beloved" Love has positive consequences for individuals. For example, studies show that love leads to improved self-esteem and self-efficacy,^2^ well-being,^3^ and creative thinking.^4^

Although love is the most wonderful and at the same time the most painful emotion that we can experience, the emotional sadness caused by the experience of the loss of love can lead to love trauma syndrome.^5^ Love trauma is defined as a set of acute symptoms that begin after the collapse of a romantic relationship and continue with the disruption of personal activity in social, professional, occupational, and academic performance.^5^ Also, since individuals with love trauma experience all or some criteria of post-traumatic stress disorder or adjustment disorder, love trauma can be also classified as trauma and stressor-related disorders (unspecified trauma and stressor-related disorder).^6-7-8-9^ On the other hand, studies have identified factors such as insecure attachment, ego and superego weakness, family problems, and anxiety problems as the causes of love trauma,^5-7-10-11-12^ and symptoms such as depression, anxiety, rumination, disturbing thoughts, arousal symptoms (such as sleep problems and irritable behavior), and adjustment problems are among its consequences.^5-6-8-13^

Another characteristic of individuals with love trauma is the feeling of helplessness and despair toward life and the future.^5^ Disappointment refers to an emotional state that is a subset of the main emotion of sadness. Disappointment greatly reduces a person's motivation and stops him from trying, and ultimately leads to depression and helplessness.^11-14^ On the other hand, the opposite of despair and in a way the antidote to despair is hope, many approaches have defined hope: hope as hopeful thinking,^15^ positive emotional experience,^16^ character strength,^17^ or a transcendental phenomenon^18-19^ but in general most theorists and authors agree that hope is positive expectations related to future consequences.^20^ An unbiased approach to hope is quoted by Dufault and Martocchio^21^ who defined hope as a multidimensional force of life that is mediated by trust and at the same time uncertain expectations of achievement about a good thing that is realistically possible and personally important for a hopeful person.

A study on stress related to growth following the loss of a romantic relationship found that those who used an optimistic interpretive style compared to those who did not use this cognitive style had experienced higher stress-related growth after the loss of a romantic relationship.^21^ This finding supports Frankel's claim that a person's attitude in certain circumstances has a definite effect on a person's ability to deal with adversity and suffering.^22^ In a recent study, Hu et al., ^23^ examined the relationship between hope, optimism, and coping strategies with post-traumatic growth. The study confirmed hope and optimism as important correlates of post-traumatic growth - with a stronger relationship between post-traumatic growth and hope than post-traumatic growth and optimism. Also, there is a significant relationship between situational hope and successful coping with problems, furthermore, situational optimism - a concept related to situational hope - is one of the most prominent predictors following a traumatic event.^16^

Variable related to the concept of hope and helplessness is positive affect and negative affect. Positive and negative affects include the emotional dimensions of personal well-being and are determined by the amount and intensity with which people are prone to touch positive and negative emotions such as happiness, excitement, anger, and sadness.^21^ Negative affect refers to the extent to which a person feels unsatisfied, it also includes the feeling of inner disappointment and not engaging in enjoyable work, which is followed by avoidant moods such as anger, sadness, and feelings of inferiority. Feelings of guilt, hatred, fear, and aggression arise. On the other hand, positive affect generally includes active energy, high concentration, and engaging in enjoyable work, followed by positive moods such as happiness, feeling empowered, enthusiasm, interest, desire, and trust. It arises naturally.^24^ Individuals with love trauma experience many emotional problems including some or all symptoms of PTSD,^8-13^ cognitive problems such as low concentration and attention, distraction and reduction of positive affects, low-level energy, and feeling of discomfort and emotional-physical lethargy.^5-6-7-10-11^

Based on studies, cognitive emotion regulation is the most effective predictor of individual mental health^25^ and includes cognitions and cognitive processes that help people regulate their emotions and feelings, this passerby should not be distracted by the intensity of emotions.^26^ Cognitive emotion regulation includes adaptive (positive) and maladaptive (negative) strategies. In the case of adaptive strategies, when faced with unfortunate events, a person blames and criticizes himself or others (self-blame/other-blame), thinks constantly about the unfortunate event (rumination), and explains it from what is considered bigger and more terrible (catastrophe). In the case of maladaptive strategies, when faced with an unfortunate event, a person probably considers related positive thoughts instead of negative thoughts (positive refocusing), giving a positive meaning to the unfortunate event (positive reappraisal), a balanced view. has a negative outlook on the event, in the sense that something worse could have happened or that something worse has happened to me before (perspective development), has a plan for personally dealing with that event (positive refocusing on planning), or that he first accepts the unfortunate event and then takes action to deal with it adaptively (acceptance).^27^

Although no study was found on the effectiveness of cognitive emotion regulation on individuals with love trauma, the practice of cognitive emotion regulation training to other harmed groups and their psychological dimensions, investigating and its effectiveness has confirmed that the effectiveness of cognitive emotion regulation training on psychological well-being,^28-29-30^ reduction of negative emotions and emotion management,^31^ positive adaptation,^32^ and reduction of symptoms of depression, anxiety, and stress^33-34-35-36^ are included. 

Also, although no study was found on the effectiveness of cognitive emotion regulation training on hope and positive and negative affects, the correlation study showed that hope has a positive and meaningful relationship with positive refocusing and positive evaluation, as well as a negative relationship and meaningful with catastrophizing^37^ and in general hope, is related to effective and efficient cognitive strategies.^38^ Also, positive emotional regulation strategies have a positive and significant relationship with positive affect and negative emotional regulation strategies have a positive and meaningful relationship with negative affect.^39-40-41^ Therefore, training cognitive emotion regulation for individuals with love trauma can be one of the practical and effective therapeutic methods based on which emotions are regulated and improved through special interventions and cognitive practices because individuals with love trauma have emotional disorders and cognitive problems.^17-42-43^ This intervention can increase compatible or positive strategies and reduce incompatible or negative strategies. 

Therefore, based on theories and studies, it is felt necessary to deal with the mental health problems of people suffering from love trauma. On the other hand, considering that, the effectiveness and efficiency of cognitive emotion regulation training have been confirmed in harmed groups, but no studies have been found in the field of the effectiveness of cognitive emotion regulation on individuals with love trauma syndrome, this research will be conducted with the aim of investigating the effectiveness of cognitive emotion regulation training based on Gross's model in improving the symptoms of love trauma, hope, and positive affect and negative affect in female students with love trauma. 

## Method

The method of this study was experimental (pre-test and post-test with the control group). The statistical population was all female students with love trauma, and bachelor's degrees, living in the dormitory of the University of Mohaghegh Ardabili. Considering that the number of samples in research with an experimental design in each group (control and experimental) is at least 15 participants^44^ and also considering the possibility of dropout of the participants, especially in the experimental group, the number of the sample was 34 participants (17 participants in each group) who will be selected by the purposeful sampling method (individuals who had a real and effective separation from a non-same-sex partner in the last 6 months). Therefore, the number of 34 female students with love trauma (selected with the LTSI) were selected and will be randomly replaced (lottery) in two experimental and control groups. Of course, the number of the initial sample was 47 participants, based on the LTSI, 35 individuals were selected, and one of them was left out randomly for matching. 

**Procedure**

After selecting the target sample and randomly replacing them in two experimental and control groups, the pre-test of three research variables (measures that include LTSI, MHS, and PANAS) was performed on both groups. Then, the cognitive emotion regulation training protocol based on the Gross model, which is a suggested method for emotion regulation training, was implemented in the experimental group. This protocol was created by James Gross (2002) to train methods of managing and regulating emotions. In this process, the control group will not receive any intervention, but after the eight training meetings for the experimental group, the measures will be performed again on both groups (post-test) to determine whether the intervention or training meetings were effective or not. Of course, due to the observance of ethical considerations after the end of the study (implementation of post-tests), the control group also received intensive training in three meetings related to the stages of cognitive emotion regulation based on the Gross model. 

**Measures**

**Love Trauma Syndrome Inventory (LTSI-10): **This 10-item measure was designed by Rosse, and its psychometric properties were good.^5^ This list measures the level of physical, emotional, cognitive, and behavioral disturbance of the sufferers on a four-point Likert scale from 0(never) to 3 (almost always). The cut-off point for the presence of love trauma is also 20.^43^ Akbari et al.^45^ reported Cronbach's alpha coefficient for this test as 0.81 and its reliability coefficient as 0.83 with a one-week interval. Cronbach's alpha in this study was also 0.78. 

**Positive Affect Negative Affect Scale (PANAS-20): **This ^46^ scale was presented by Watson, Clark, and Tellgen^46^ in 20 items that express twenty feelings or affects. Meanwhile, there are ten positive affects: interest, enthusiasm, strength, pride, honor, vigilance, cleverness, tastefulness, determination, awareness, and precision. On the other hand, the scale measures ten negative affects, including anxiety, discomfort, guilt, fear, irritability, shame, restlessness, anger, and fear, on a five-point range from 1 (strongly disagree) to 5 (strongly agree). Also, the range of scores for positive affect and negative affect is 10 to 50. The psychometric properties of the main scale have been reported favorably.^46^ Bakhshipour Rudsari and Dezhkam also reported that the internal consistency coefficient (alpha coefficient) for both dimensions of the scale (positive affect and negative affect) was 0.85.^47^ Cronbach's alpha in this study was also 0.81. Cronbach's alpha in this study was 0.79 for positive affect and 0.77 for negative affect. 

**Miler Hope Scale (MHS-48):**This scale was created by Miller and Powers^48^ and was first used to measure the hope of heart patients in the United States of America to evaluate their level of hope. This scale is a type of diagnostic test and includes 48 aspects of states of hope and helplessness, whose items are selected based on overt or hidden behavioral manifestations in hopeful or helpless (hopeless) people. Miller has reported the psychometric properties of this scale as favorable.^48^ The items are scored on a five-point Likert scale from 1(strongly disagree) to 5 (strongly agree). The subject's score can range from 48 to 240. A score of 48 indicates that the person is completely helpless and a score of 240 indicates that the person is completely hopeful.^49^ Malek Afzali also^50^ reported Cronbach's alpha reliability of 0.91 for this scale. In this study, Cronbach's alpha was 0.81. 

**Cognitive emotion regulation intervention program based on Gross model: **This educational program is called the Gross model, which was proposed by Gross in 2002. It is an intervention method for emotion regulation, and involves eight group sessions that are each one-and-a-half hours long. The meetings of the cognitive emotion regulation training intervention program are described in the Table A.

**Statistical analysis**

The statistical methods used in the descriptive statistics section were mean and standard deviation, and in the inferential statistics section, if the conditions are met it is multivariate covariance analysis (MANCOVA). The gender of all participants was female and their average age was 21 years. They were students studying at the undergraduate level at the University of Mohaghegh Ardabili. Also, one person who had a large variance with other subjects was removed from each group (control and experiment).

**Table A:** Topics, targets and content of cognitive emotion regulation training meetings based on the Gross model.

Meetings (1-8)	Targets	The content of the meetings
Introduction	getting to know about gross model-based cognitive emotion regulation training	i) Introducing the group members to each other and starting the mutual relationship between the group leader (consultant) and the members, ii) Expressing the main and secondary goals of the group and discussing the personal and collective goals of the members, and iii) Expressing the logic and Stages of cognitive emotion regulation training (intervention).
Skills of understanding emotion and choosing a situation	providing emotional training	i) Recognition of emotions and situations that provoke emotions, ii) Presentation of information about different dimensions of emotions and different emotions, as well as short-term and long-term effects of emotions, and iii) Emotional self-awareness.
Prevention	Evaluation of vulnerability and emotional skills of members	i) Recognition of emotional experiences based on self-evaluation, ii) Level of emotional vulnerability based on self-evaluation, and iii) Identification of regulatory strategies based on self-evaluation.
Correction of position	Making a change in the exciting situation	i) Preventing social isolation and avoidance, ii) Teaching problem-solving strategies, and iii) Teaching interpersonal skills (conversation, self-expression and conflict resolution).
Expansion of attention	Attention shift	i) stop rumination and worry, and ii) train attention.
Cognitive evaluation	Changing cognitive evaluations	i) The role of cognition in producing, maintaining, reducing and increasing emotional responses, ii) Identifying incorrect evaluations and their effects on emotional states, and iii) Teaching re-evaluation strategies.
Adjusting the target response	Changing the behavioral and physiological consequences of emotion	i) Identifying the amount and method of using the inhibition strategy and examining its emotional consequences, ii) Exposure: creating emotional states, iii) Teaching emotion expression, iv) Modifying behavior. Through the change of environmental reinforcers, and v) emotional discharge training, relaxation and reverse action.
Evaluation and practice	Re-evaluate and remove barriers	i) Evaluation of the achievement of individual and group goals, ii) practice of learned skills in external environments, and iii) Examining and removing obstacles to completing assignments and teachings.

## Results

The final analysis was done on two groups with 16 participants. Table 1 shows the descriptive findings including the mean and standard deviation of the subjects in the two experimental and control groups, in the pre-test and post-test, and the total score.

**Table 1:** Descriptive statistics of love trauma syndrome variables, hope, and positive affect negative affect.

Variables	Groups	pre average	Standard deviation test	average	Standard deviation test
Love trauma syndrome	the experiment	20.63	3.292	8.25	3.059
	control	20.88	2.642	21.50	3.928
	Total	20.75	2.887	14.88	7.641
Hoping	the experiment	161	23.628	189	13.732
	control	166.13	28.886	150.88	31.421
	Total	163.56	25.631	169.94	30.599
Positive affect	the experiment	30.25	7.146	37.88	4.853
	control	38.25	9.925	31	8.315
	Total	34.25	9.320	34.44	7.474
Negative affect	the experiment	33.25	4.803	20.25	3.955
	control	35	6.969	33.75	5.312
	Total	34.13	5.852	27	8.311

In terms of demographic data, all the participants were in the third and fourth years of the bachelor's degree (educational sciences and psychology), in terms of socio-economic status in the middle level of society, and in terms of age, they were between 20 and 22 years old.

Table 1 shows the descriptive statistics, including pre-test and post-test averages and standard deviations in the experimental and control groups.

Table 2 shows the homogeneity of covariances, which according to the significance level (p>0.05), this assumption is met and there is no problem in terms of the homogeneity of covariances. Also, Table 3 shows the homogeneity of variances, which according to the significance level (p>0.05), this assumption is also met and there is no problem in terms of homogeneity of variances.

**Table 2:** M-box test for homogeneity of covariances.

Source	Box’s M	F	Degree of freedom 1	Degree of freedom 2	Significance level
Group	5.050	1.423	3	35280.000	0.234

**Table 3:** Levine's test for homogeneity of variances.

Variables	F	Degree of freedom 1	Degree of freedom 2	meaningful
Love trauma syndrome	1.109	1	30	0.310
Hoping	2.253	1	30	
Positive affect	1.109	1	30	0.310
Negative affect	2.253	1	30	0.159

Table 4 or the results of the multivariate analysis of covariance (MANCOVA) shows that there is a significant difference between the groups in terms of variables (p<0.01).

**Table 4:** The results of multivariate analysis of variance (MANCOVA) for the variables of love impact, hope, and positive affect negative affect.

Source	Test	Value	F	df hypothesis	df error	Significance level	Partial Eta Squared
	Pillai trace	0.804	22.595	2	11	0.001	0.804
Group	Wilks Lambda	0.196	22.595	2	11	0.001	0.804
	Hotling trace	4.108	22.595	2	11	0.001	0.804
	Roy's Largest Root	4.108	22.595	2	11	0.001	0.804

As can be seen in Table 5, before the intervention, there was no significant difference between the groups in the variables of love trauma syndrome, hoping, positive affect, and negative affect, (p>0.05), but after the intervention, there was a significant difference between the groups in the variables of love trauma syndrome, hoping, and negative affect (p<0.01). Based on the explanation that the love trauma syndrome and negative affects decreased in the experimental group and hope increased in them, but these changes did not occur in the control group., as seen in Table 1.

**Table 5:** Test of differences between groups in the variables of love, hope, and positive and negative affect.

Source	The dependent variables	sum of squares	Degrees of freedom	mean square	F	Significance level	Partial Eta Squared
Pre-test effect	Love trauma syndrome	0.444	1	0.444	0.31	0.863	0.003
	Hoping	2.507	1	2.507	0.005	0.947	0.001
	Positive affect	6.134	1	6.134	0.116	0.739	0.010
	Negative affect	11.841	1	11.841	0.522	0.484	0.042
The effect of group membership	Love trauma syndrome	700.281	1	700.281	49.101	0.001	0.804
	Hoping	6019.516	1	6019.516	11.019	0.006	0.479
	Positive affect	188.380	1	188.380	3.567	0.083	0.229
	Negative affect	704.885	1	704.885	31.074	0.001	0.721

## Discussion

Based on the Gross model, cognitive emotion training can reduce the severity of love trauma syndrome. Related research suggests that trauma-focused cognitive behavioral therapy can also help.^51^ Additionally, Rajabi, Tabnak, and Nazarpour in a study^52^ showed that individuals with experiencing love trauma have difficulty using cognitive emotion regulation strategies, aside from broadening the perspective. Since cognitive emotion regulation has many trauma symptoms (such as flashbacks or insomnia) and is linked to harm,^26^ using adaptive strategies can act as a trauma absorber and reduce harm. Therefore, correcting the maladaptive strategies and strengthening the adaptive strategies leads to the reduction of harm. 

As a result, the afflicted individual may eventually acquire a healthier and more balanced viewpoint on things and a load of a negative impact can be lessened for several reasons: i) applying adaptive strategies and thoughts that can reduce negative and catastrophic thoughts and foster positive expectations, however low, in a person; ii) correcting incompatible thoughts like zooming in and out; iii) collectivism and the feeling that individuals like me have experienced separation and emotional failure (group training and collective situational effects). Of course, in this direction and during the meetings, the harmed person repeatedly receives empathetic feedback from the group members and the group leader, and this process can also have a positive and healing effect so that the person in a more appropriate affective relationship can achieve better cognitive and social achievements. For example, a healthier perspective and distance from isolation. 

Also, the results showed that cognitive emotion regulation training based on the Gross model will lead to an increase in hope. Although no direct and similar studies were found in this field, a close study showed that positive refocusing, positive evaluation, and positive cognitive regulation have a positive relationship with hope, and catastrophizing has a negative relationship with it. On the other hand, positive cognitive regulation has a positive and meaningful relationship with the active thinking component. Also, catastrophizing and rumination have a positive relationship with depression, and positive evaluation has a negative relationship with depression.^39^

When the maladaptive strategies of catastrophizing and rumination that naturally lead to helplessness (or despair) are activated in a person, the correction of these maladaptive strategies through cognitive feedback is needed. Therefore, this feedback and training in the form of models can be an effective strategy. In this context, four educational dimensions are more important: i) expansion of attention including learning to return attention and clear verbal feedback to stop rumination (such as stop or enough), ii) cognitive evaluation includes identifying incorrect evaluations and their effects on emotional states and then teaching re-evaluation strategies to correct grandiosity and catastrophizing (for example., asking the affected person to state evidence about Experiencing bitter events in the past or the love trauma that happened to friends and peers, then comparing them with the current situation), iii) Exaggerating the description of events with high excitement and the gradual decline of the same excitement in the context of the passage of time, and iv) Modifying the maladaptive strategies of catastrophizing and rumination. These cases lead the sufferer to the flexibility of dry thoughts and reduce the intensity of the negativity of the incident and regrets, which ultimately leads to a decrease in helplessness (despair) and an increase in hope. Therefore, following changes and cognitive patterning, hope and optimistic expectations gradually replace helplessness and dry and negative thoughts. 

The results showed that cognitive emotion regulation training based on the Gross model will not lead to a change in positive affect (increase). Also, no studies were found in this field. Positive affect includes interest, enthusiasm, strength, enthusiasm, pride and honor, alertness and cleverness, good taste, being determined, being precise and aware, and being active. It seems that the harmed individuals, especially those who suffer from external events such as love trauma, have significant affective and behavioral problems, in addition to the need for clinical interventions to resolve maladaptive cognitions and reduce pathological symptoms need time to get more effective and efficient. In the meantime, the passage of time, in addition to reducing the severity of pathological symptoms and reducing emotions, can make the interventions more suitable from an individual point of view and gradually increase the recovery and ultimately the efficiency.^53^ Of course, this issue is more relevant for situational harms such as bereavement and love trauma, and in the case of serious harms (such as PTSD), short-term interventions, and release cannot meet the needs of patients. Also, considering that individuals with severe situational harms (such as love trauma syndrome), usually have other more fundamental harms (such as attachment and personality problems).^7-11^ Therefore, it seems reaching the desired efficiency point requires individual psychotherapies to be more serious, at least for some of them.

On the other hand, the results showed that cognitive emotion regulation training based on the Gross model will lead to a reduction in negative affects. No studies were found in this field. Negative affects include anxiety, discomfort, guilt, fear, irritability, shame, restlessness, anger, and fear. Individuals with love trauma syndrome experience some negative affects (such as restlessness, guilt, and sadness).^7^ All cognitive maladaptive strategies include blaming oneself, blaming others, rumination, and catastrophizing along with the negative affects of losers.^45^

Although the relationship between negative affects and maladaptive strategies can be two-way, in cognitive approaches, cognition precedes effects and emotions. Therefore, according to the correction of incompatible strategies in the sessions of cognitive emotion regulation and the replacement of adaptive cognitions with them, negative affects were reduced. In this context, on the one hand, cognitive evaluation by identifying incorrect evaluations and their impact on emotional states, as well as teaching re-evaluation strategies; on the other hand, modifying the target's response with self-expression training, behavior modification through environmental reinforcers, and emotional discharge intervened in projecting, reducing and eliminating negative affects. Of course, it seems that the path followed in the meetings means i) acceptance and non-confrontation with resentment, ii) emotional discharge and release of negative affective accumulations, and finally, iii) identification and correction of maladaptive cognitions. Three steps were effective in reducing negative affects. 

A significant impact on one group of affects (including negative affect) does not necessarily mean an impact on another group (including positive affect). In the explanation of this finding, it can be said that positive affect includes the tendency to experience intense pleasant feelings, in its upper pole there is excitement and happiness, and in its lower pole these feelings are absent and do not exist, but there are not necessarily negative affects. On the other hand, negative affect is the tendency to experience intense unpleasant feelings, and at its upper pole there are feelings such as anger, and anxiety, and at its lower pole there are no such feelings, but there are not necessarily positive affects. Also, many pieces of evidence show that positive and negative affects have nothing to do with each other and are not two sides of the same coin. This is because positive affects are generated by pleasant events and experiences, while negative affects are caused by unpleasant events.^54^

This study had limitations. The sample was only from one university, one age group, and one gender. Therefore, future studies can be conducted in other age groups (e.g., teenagers) and males (boys). Also, the period of separation (six months or more) and paper-pencil tools were among other limitations. Therefore, a shorter separation period for the target sample and the implementation of clinical interviews, before and after the intervention, along with quantitative tools, make the data obtained from the participants more accurate and detailed. Based on the results, the intervention of cognitive emotion regulation training, based on the Gross model, can be implemented by counselors and therapists in the form of counseling and group training in universities and schools to improve more individuals with love trauma syndrome because this intervention by increasing adaptive cognitive strategies (such as positive reappraisal) and reducing maladaptive strategies (such as rumination) in traumatized individuals gradually improves harms (such as love trauma syndrome) and cultivates positive aspects in them (such as hoping). 

**Acknowledgements**

The research department of Mohaghegh Ardabili University and all study participants are greatly appreciated by the authors of this article.
